# Inhibiting the Growth of Pancreatic Adenocarcinoma *In Vitro* and *In Vivo* through Targeted Treatment with Designer Gold Nanotherapeutics

**DOI:** 10.1371/journal.pone.0057522

**Published:** 2013-03-06

**Authors:** Rachel A. Kudgus, Annamaria Szabolcs, Jameel Ahmad Khan, Chad A. Walden, Joel M. Reid, J. David Robertson, Resham Bhattacharya, Priyabrata Mukherjee

**Affiliations:** 1 Department of Biochemistry and Molecular Biology, College of Medicine, Mayo Clinic, Rochester, Minnesota, United States of America; 2 Department of Physiology and Biomedical Engineering, College of Medicine, Mayo Clinic, Rochester, Minnesota, United States of America; 3 Department of Chemistry and University of Missouri Research Reactor, University of Missouri, Columbia, Missouri, United States of America; 4 Mayo Clinic Cancer Center, College of Medicine, Mayo Clinic, Rochester, Minnesota, United States of America; Children’s Hospital Boston & Harvard Medical School, United States of America

## Abstract

**Background:**

Pancreatic cancer is one of the deadliest of all human malignancies with limited options for therapy. Here, we report the development of an optimized targeted drug delivery system to inhibit advanced stage pancreatic tumor growth in an orthotopic mouse model.

**Method/Principal Findings:**

Targeting specificity *in vitro* was confirmed by preincubation of the pancreatic cancer cells with C225 as well as Nitrobenzylthioinosine (NBMPR - nucleoside transporter (NT) inhibitor). Upon nanoconjugation functional activity of gemcitabine was retained as tested using a thymidine incorporation assay. Significant stability of the nanoconjugates was maintained, with only 12% release of gemcitabine over a 24-hour period in mouse plasma. Finally, an *in vivo* study demonstrated the inhibition of tumor growth through targeted delivery of a low dose of gemcitabine in an orthotopic model of pancreatic cancer, mimicking an advanced stage of the disease.

**Conclusion:**

We demonstrated in this study that the gold nanoparticle-based therapeutic containing gemcitabine inhibited tumor growth in an advanced stage of the disease in an orthotopic model of pancreatic cancer. Future work would focus on understanding the pharmacokinetics and combining active targeting with passive targeting to further improve the therapeutic efficacy and increase survival.

## Introduction

Pancreatic cancer is the fourth leading cause of cancer deaths in America [1]. It continues to have a less than 5% survival rate over 5 years, with a median survival of only six months [2], [3]. Pancreatic cancer is an aggressive and illusive cancer that is typically diagnosed at the late stages of the disease where surgical intervention is no longer an option and traditional chemotherapeutics have minimal therapeutic effects.

Gemcitabine is the standard of care for pancreatic cancer treatment [4]–[8]. The therapeutic efficacy of gemcitabine is governed by the triple phosphorylation within the cell by deoxycytidine kinase (dCK) to an active form; followed by subsequent intercalation into the DNA of the cell leading to the inhibition of DNA synthesis and hence, inhibition of cellular proliferation [9], [10]. Despite this being the current protocol, gemcitabine continues to have a modest beneficial outcome on its own in a clinical setting [11], [12]. There have been a number of combination therapies that utilize gemcitabine and other drugs or antibodies in an attempt to enhance the therapeutic effects of the gemcitabine, but all have shown dismal outcomes [4], [13]–[17].

Nanotechnology has the potential to overcome the limitations in current cancer therapeutic options [18]–[23]. The adverse effects of chemotherapies are an enormous problem in the current treatment of cancer in general, causing systemic toxicity leading to severe side effects. The utilization of monoclonal antibodies conjugated to gold nanoparticles has proven to be effective in targeting cancer cells with an over expression of EGFR (epidermal growth factor receptor) [24], [25]. Gold nanoparticles have been shown to be biologically viable and highly adaptable for conjugation with nearly any compound having an amine or thiol functionality utilizing Au-SH, Au-NH_2_ interactions [18], [26]–[30].

In this study we utilized cetuximab, an anti-EGFR monoclonal antibody, as a targeting agent. Cetuximab was approved by the FDA for the treatment of colorectal cancer, as well as head and neck cancer in 2004 [31]–[38]. Our group previously reported the effective targeting of EGFR-overexpressing pancreatic cancer cells both *in vitro* and *in vivo* with gold nanoparticles conjugated with C225 as a targeting agent [24]. Utilizing these findings we now incorporated gemcitabine in the nanoformulation to create an optimized targeted drug delivery vehicle to inhibit the growth of pancreatic cancer cells simulating an advanced stage of the disease in an orthotopic model.

The aim of this current study was to develop a gold nanoparticle-based therapeutic with enhanced efficacy to inhibit pancreatic cancer growth in an advanced stage of the disease. The designer therapeutic introduced in this paper is a novel approach to increasing the efficacy of gemcitabine, or any chemotherapy, with the utilization of a targeted delivery system that employs gold nanoparticles as the delivery vehicle. This study was aimed to evaluate the *in vitro* and *in vivo* anti-tumor effect of a gold nanoparticle based targeted drug delivery system that inhibits pancreatic tumor growth in an advanced stage of the disease.

## Materials and Methods

### Materials

Tetrachloroauric acid trihydrate and sodium borohydride were from Sigma-Aldrich, St. Louis, MO. ^3^H-thymidine was from Perkin-Elmer, (Waltham, MA). Media and PBS was purchased from Mediatech (Manassas, VA). Scintillation cocktail was purchased from Fisher Scientific.

### Synthesis and Characterization of Au-antibody and Au-antibody-gemcitabine Nanoconjugates

The core gold nanoparticles (GNPs) were synthesized by reduction of 1200 ml of 0.1 mM tetrachloroauric acid trihydrate (HAuCl_4_) solution with 600 ml of a freshly prepared aqueous solution containing 51.6 mg of sodium borohydride (NaBH_4_) under vigorous and constant stirring, overnight at ambient temperature. Upon addition of the sodium borohydride, the pale yellow solution becomes orange and then turns to a wine red color within minutes. The GNPs were characterized by UV-visible spectrometry, scanning from 400–800 nm and transmission electron microscopy (TEM) after drop-coating 10 µl of the sample on a 400 mesh carbon-coated copper grid followed by side blotting. The size of the nanoparticles was determined from analysis of the TEM images and Dynamic Light Scattering (DLS) (Malvern Zetasizer Nano ZS). Zeta potential measurements were done using a clear zeta disposable capillary (Malvern DTS1061).

The GNP-antibody conjugates (AC4 and AI4) were synthesized by mixing 4 µg/ml of antibody (C225 or IgG, respectively) with the core GNP solution as previously reported. Cetuximab (C225) was purchased as a solution of 2 mg/ml (Erbitux™ Injection, ImClone Inc and Bristol-Myers Squibb Co.) and whole molecule human IgG was purchased as a solution of 10.0–11.2 mg/ml (Jackson Immuno Research Laboratories, Inc.). After dilution in 1 ml of water each antibody was added dropwise to the GNP solutions. These solutions were stirred vigorously at ambient temperature for 1 hr. One half of these solutions were centrifuged at 20,000 rpm in a Beckman Ultracentrifuge in a 50.2 Ti rotor to separate AC4 and AI4 nanoconjugates from unconjugated antibody.

The other half of the solution was subjected to further conjugation with various concentrations of gemcitabine (Eli Lilly, Indianapolis, IN) to generate ACG4X and AIG4X (X = 1, 2, 4, 6 and 8 µg/ml). These solutions were also stirred vigorously at ambient temperature for 1 hr and then centrifuged at 20,000 rpm in a Beckman Ultracentrifuge in a 50.2 Ti rotor to separate ACG4X and AIG4X from unbound antibody and gemcitabine. All conjugates formed a loose pellet at the bottom of the centrifuge tube and were collected after careful aspiration of the supernatant. The gold concentration of the nanoconjugates was determined from absorbances obtained by UV-visible spectrometry (SpectraMax M5e) at 500 nm (A_500_) and 800 nm (A_800_), taken before and after centrifugation and by instrumental neutron activation analysis (INAA). The antibody loading was previously determined [24] and the gemcitabine concentration in the nanoconjugates was determined through high-performance liquid chromatography (HPLC) analysis of the supernatant and subtracted from the total µg added to determine the bound concentration as previously reported [25]. The size and hydrodynamic diameter of the nanoparticle conjugates was determined from analysis of the TEM images and DLS, respectively. Zeta potential measurements were done using a clear zeta disposable capillary (Malvern DTS1061).

The stability of the ACG4X and AIG4X conjugates was tested against 150 mM sodium chloride (NaCl) solution. The absorbance spectrum was taken for all nanoconjugates before and after incubation with NaCl solution for 15 minutes.

### Release Study

The release profile of gemcitabine in different biological fluids was characterized following incubation of 100 µl of nanoconjugates having a gemcitabine concentration of 24.4 µg/ml with 100 µl of PBS or mouse plasma and incubated for different time periods. For a control, nanoconjugates were incubated in water, this sample was used to determine the baseline (free) gemcitabine concentration at the zero time point. The samples were centrifuged at 100,000 g for 1 hr and the supernatants were collected. The concentration of gemcitabine in the supernatants was determined by HPLC analysis. The total concentration of gemcitabine at different time points was plotted after subtracting the baseline concentration.

### Cell Culture

Pancreatic cancer cell lines: AsPC-1, PANC-1 and MiaPaca-2 were grown in RPMI and Dulbecco’s Modified Eagles medium (Gibco). All media was supplemented with 10% fetal bovine serum (Gibco) and 1% antibiotics (Penicillin/Streptomycin) and the cell lines were maintained at 37°C, in a humidified atmosphere under 20% O_2_ and 5% CO_2_.

### ^3^H-thymidine Incorporation Assay

For each cell line, (3×10^4^) cells were seeded in 24-well culture plates in their respective media and allowed to incubate overnight (16 hrs). After incubation for 2 hrs with ACG44, AIG44 and no gold controls at 0.1, 1 and 10 µM concentrations of gemcitabine cells were washed with PBS to remove unbound nanoconjugates and replaced with fresh media followed by additional incubation for another 48 hrs. At the end of 48-hr incubation the experiment, the media in the wells was replaced with ^3^H-thymidine containing media (1 µCi/mL) and incubated for an additional 4 hrs at 37°C and processed for the assay as described previously [39]. Experiments were repeated at least three times, in triplicate each time, averages and standard deviations are reported.

### In vitro Targeting Studies

To determine the effect of serum components on the targeting efficacy of the nanoconjugates, we preincubated AC4, AI4, ACG44 and AIG44 in RPMI containing fetal bovine serum for 15 mins. After incubation we studied cellular uptake in AsPC-1 cells in 100 mm tissue culture dishes. Both preincubated and as synthesized nanoconjugates were added to the cells in separate culture dishes and incubated at 37°C for 2 hrs. After the treatment the media was removed and the cells were washed once with PBS to remove excess nanoconjugates and trypsinized to obtain a cell pellet for gold content determination by INAA.

### Inhibitor Studies

Cells were pre-treated with either C225 or NBMPR (Nitrobenzylthioinosine) to determine the specificity of uptake of ACG44 and AIG44 nanoconjugates. AsPC-1 cells were grown in 100 mm tissue culture dishes and pre-treated with either 50 µg/mL of C225 or 100 nM NBMPR for 1 hr followed by the addition of 2 µg/mL ACG44 and AIG44, respectively. After 2 hrs, the culture medium was removed, the plates were washed once with PBS to remove any unbound nanoconjugates and cells were trypsinized to obtain a cell pellet to determine gold content by INAA.

### Measurement of Gold Content by Instrumental Neutron Activation Analysis (INAA)

Samples were analyzed by instrumental neutron activation analysis at the University of Missouri Research Reactor Center as previously described [24], [25], [40]–[43]. Briefly, cell pellets/tissues were prepared by weighing the samples into high-density polyethylene irradiation vials and lyophilized to a dry weight. Solution samples were prepared by gravimetrically transferring 100 µl to an irradiation vial followed by lyophilization. All samples were loaded in polyethylene transfer “rabbits” in sets of nine and irradiated for 90 sec in a thermal flux density of approximately 5×10^13^ n cm^−2^ s^−1^. The samples were then allowed to decay for 24 to 48 hrs and counted on a high-purity germanium detector for 3600 sec at a sample-to-detector distance of approximately 5 cm. The mass of gold in each sample was quantified by measuring 411.8 keV gamma ray from the β^-^ decay of ^198^Au (t_1/2_ = 2.7 days). The area of this peak was determined by the Genie ESP spectroscopy package from Canberra. A minimum of six geometrically equivalent comparator standards were also run. The standards were prepared by aliquoting approximately 0.1 (n = 3) and 0.01 (n = 3) µg of gold from a (10.0±0.5) µg/mL certified standard solution (High-Purity Standards) in the polyethylene irradiation vials, and were used with each sample set.

### Transmission Electron Microscopy (TEM)

TEM samples (cell pellets and tissues) were fixed in trumps solution and processed as previously described [24]. Micrographs were taken on a TECNAI 12 operating at 120 KV.

### Animal Handling and in vivo Tumor Uptake

Male athymic nude mice (NCr-nu; 4–6 weeks old) were purchased from the National Cancer Institute-Frederick Cancer Research and Development Center (Frederick, MD). All mice were housed and maintained under specific pathogen-free conditions in facilities approved by the American Association for Accreditation of Laboratory Animal Care and in accordance with current regulations and standards of the U.S. Department of Agriculture, U.S. Department of Health and Human Services, and NIH. All studies were approved and supervised by the Mayo Clinic Institutional Animal Care and Use Committee.

For the generation of orthotopic pancreatic tumor models, before injection, tumor cells were washed twice with PBS, lifted with 0.25% trypsin, centrifuged for 5 minutes, and reconstituted in PBS. AsPC-1 cells (1.5 × 10^6^) were implanted into the pancreas of nude mice. The mice were imaged non-invasively every week under isoflurane anesthesia using a Xenogen-IVIS-cooled CCD optical system (Xenogen-IVIS) as previously described [24]. Seven days after tumor cell implantation, the mice were randomized into 6 groups (n = 5). All nanoconjugates were normalized to a gold concentration of 450 µg/mouse (1.8 mg/kg of gemcitabine) and injected i. p. thrice for week one and twice for weeks two and three. The mice were sacrificed on day 28 and the tumors, kidneys, liver, lungs, spleen, pancreas and blood were collected and analyzed for gold content through INAA.

### Immunohistochemistry

Tumor samples were fixed in 4% paraformaldehyde and stained for hemotoxylin and eosin (H&E) and Ki-67. The percentage of Ki-67 positive cells in 5 high-powered fields at 20X was determined through visual inspection and counting.

### Statistical Analysis

Statistical analysis was done by a two-tailed student t-test and a value of P<0.05 was considered to be significant.

## Results and Discussion

### Synthesis and Characterization of Gold Nanoparticles and Nanoconjugates

Physicochemical characterization of the unmodified GNPs and the nanoconjugates were performed by UV-visible spectroscopy (UV-vis), transmission electron microscopy (TEM), dynamic light scattering (DLS) and zeta potential (ζ-Potential) measurements.

The UV-visible spectrum of unmodified GNPs exhibits a characteristic surface plasmon resonance (SPR) band of spherical gold nanoparticle at 512 nm as previously reported [24], [25]. Addition of an anti-EGFR antibody cetuximab (C225), or its non-targeted counterpart immunoglobulin G (IgG) at a concentration of 4 µg/ml to the GNP solution increases the absorbance of the solution with a simultaneous red shift in the λ_max_ value from 512 nm of unmodified GNP to 518 nm for Au-C225 (AC4) and Au-IgG (AI4) conjugates [24], [25], respectively. Such a red shift in the SPR band suggests binding of C225 and IgG to the GNP surface as previously reported [24], [44]. The rationale for selecting a concentration of 4 µg/ml of C225/IgG was based on our previous report where we demonstrated that the AC4 conjugates have the highest ability to target EGFR-overexpressing pancreatic cancer cells both *in vitro* and *in vivo* in an orthotopic pancreatic cancer model [24]. Based on this information, we next designed a targeted drug delivery system with the incorporation of gemcitabine (Gem) on the AC4 and AI4 nanoconjugates. The addition of different concentrations of gemcitabine (1, 2, 4, 6, and 8 µg/ml) to the AC4 and AI4 solutions caused a further red-shift in the λ_max_ from 518 nm to 520 nm for the ACGs and AIGs, suggesting binding of gemcitabine to the available reactive surface on the gold nanoparticle (Figures S1A,B).

**Figure 1:**
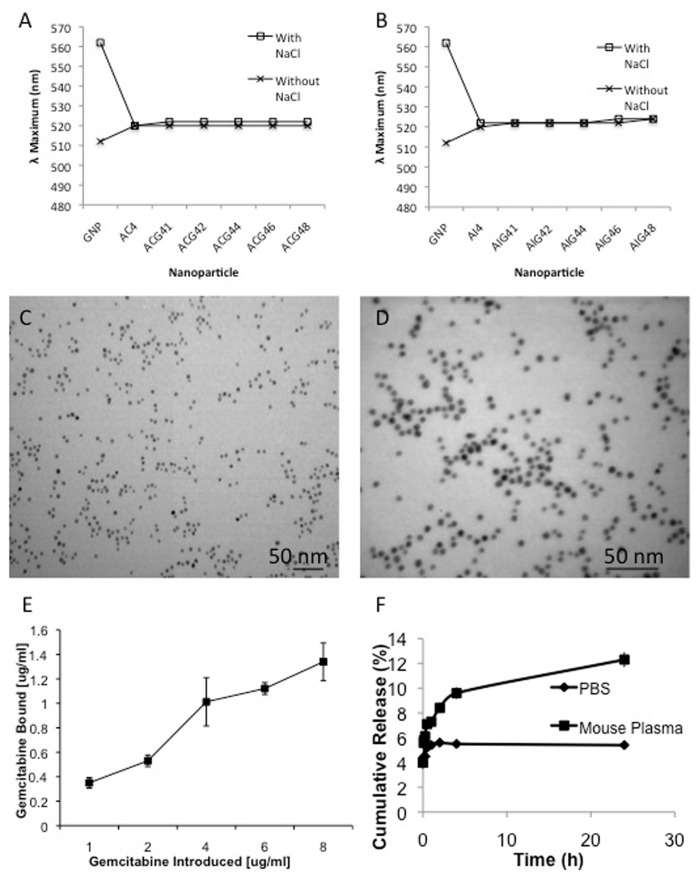
Physicochemical characterization of gold nanoconjugates. Figure 1A and 1B describes the changes in the λ_max_ value of GNP and different ACG44 and AIG44 nanoconjugates, respectively, with/without incubation with NaCl for 15 minutes. Figure 1C and 1D exhibits the transmission electron micrographs (TEM) of ACG44 and AIG44 nanoconjugates, drop coated after synthesis without alteration. Figure 1E describes the amount of gemcitabine bound to AC4 nanoconjugates analyzed by HPLC; the x-axis shows the µg/ml used to synthesize the conjugates and the y-axis represents the µg/ml bound to the particle. Figure 1F describes the release of gemcitabine from ACG44 nanoconjugates when incubated in PBS and mouse plasma over time.

Stability of the nanoconjugates in 150 mM sodium chloride (NaCl) further supports the binding of both C225/IgG and gemcitabine on the GNPs. It is evident from Figure 1A and B that the addition of 150 mM NaCl to unmodified GNPs shifted the λ_max_ from 512 nm to 562 nm with a strong decrease in absorbance, suggesting significant aggregation of uncovered GNPs by NaCl in the absence of surface protection by C225/IgG and Gem. However, the addition of NaCl to the ACGs and AIGs did not alter the absorbance and λ_max_ value, thereby confirming the stabilization of GNPs by conjugation with C225/IgG and gemcitabine. TEM analysis further confirms the absence of aggregation as well as the formation of ∼5 nm spherical nanoparticles (Figure 1C, D). Dynamic light scattering and zeta potential results were used to further characterize the hydrodynamic diameter (HD) as well as the charge of the particles at each step of conjugation (Table 1 and Table S1).

**Table 1:** Dynamic Light Scattering and Zeta Potential Measurements of GNP (the core particle), ACG44 and AIG44.

Sample	DLS (d.nm)	Zeta Potential (mV)
GNP*	5.3	−29.6
ACG44	32.09	−20.2
AIG44	88.95	−20.6

A MELVERN Zetasizer Nano ZS instrument was used to measure both the DLS and ζ-Potentials of all the nanoconjugates. The average of 5 independent runs is presented in Table 1. As expected, the HD increases from 5 nm for the unmodified GNP, to approximately 38 nm for AC4 and 56 nm for AI4, further demonstrating the binding of C225 and IgG to GNP. The addition of gemcitabine, at all concentrations does not alter the HD of the nanoconjugates; this observation was expected due to the small molecule size of gemcitabine. The ζ-potential measurements also followed a similar trend; as expected, the GNP was most negative, with an average of approximately −30 mV. The ζ-Potential starts to become less negative with the addition of C225 and IgG due to the antibody binding. In turn, there is a minor increase in ζ-potential with the addition of gemcitabine. These results clearly suggest the binding of C225/IgG and gemcitabine to the nanoparticle to form ACGs and AIGs.

### Quantifying the Gemcitabine Loading on AC4 and AI4

Using varying concentrations of ^125^I-labeled C225 and IgG we previously demonstrated that nearly 90% of 4 µg/ml antibody was bound to the GNP [24]. To determine the loading of gemcitabine on AC4 and AI4, gemcitabine was added to the antibody covered particles in various concentrations (X = 1, 2, 4, 6 and 8 µg/ml) under vigorous stirring at room temperature. After one hour, the nanoconjugates containing gemcitabine were purified by ultracentrifugation. The ACG4X and AIG4X pellets were collected at the bottom of the centrifuge tubes and the supernatant, containing the unbound gemcitabine was analyzed using HPLC. The bound fraction was calculated by subtracting the concentration of gemcitabine present in the supernatant from the original concentration introduced during the nanofabrication process. The concentration of bound gemcitabine increases from approximately 0.4 to 1 µg/ml for the ACG4X particles with the introduction of 1–4 µg/ml (Figure 1E). However, the addition of 6 and 8 µg/ml of gemcitabine does not significantly increase the amount gemcitabine in the nanoconjugate. Therefore, the optimum conjugation reaction with the highest percent yield for gemcitabine bound to the particle was determined to be 4 µg/ml. As expected, the AIG4X particles showed a similar trend for gemcitabine binding (data not shown). Subsequently, the ACG44 and AIG44 nanoconjugates were employed for all further experiments.

The ACG44 and AIG44 nanoconjugates were synthesized using the AC4 and AI4 solutions, respectively, as described in the materials and methods section. This synthesis was possible due to the spontaneous binding of the antibodies through their cysteine/lysine residues utilizing the Au-S/Au-NH_2_ bond as previously described [24]. It has been well described in the literature that interactions of proteins, antibodies and small molecules with gold could be due to electrostatics, covalent bonding or hydrophobic interactions. The addition of gemcitabine exploits the Au-NH_2_ binding through the amine moiety on gemcitabine to the gold particle surface. Our previous data suggests the initial binding is due to electrostatics and then covalent, investigated with XPS and TGA analysis and previously reported by our group [25], [45].

### Mechanism and Targeting Efficacy of ACG44 and AIG44 in vitro

**Figure 2:**
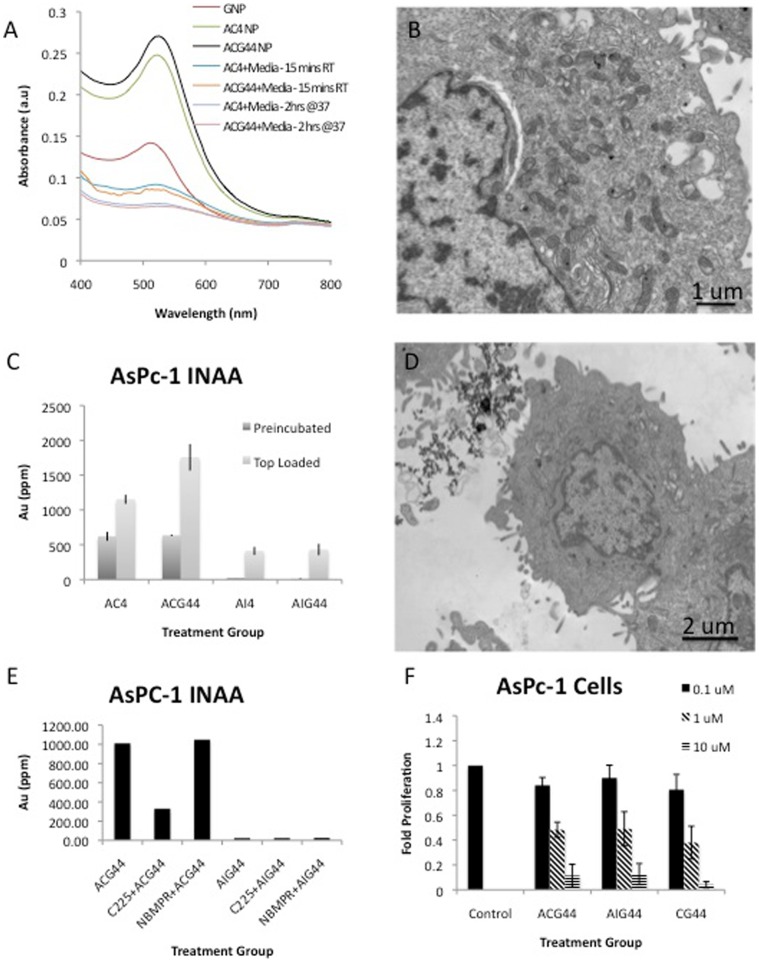
Role of pre-incubation with serum, C225 and NBMPR on targeting efficacy of gold nanoconjugates and their *in vitro* biological function. Figure 2A depicts the absorbance spectrums of GNP, AC4 and ACG44 before and after pre-incubation with serum either 15 minutes at room temperature or 2 hrs at 37°C. Figure 2B and 2D are transmission electron microscopy images of the *in vitro* uptake of ACG44 and AIG44 in AsPC-1 cells, respectively. Figure 2C describes the effect of pre-incubation with serum on the cellular uptake of the nanoconjugates into AsPC-1 cells analyzed for gold content utilizing INAA. Figure 2E also depicts INAA analysis of cellular uptake of ACG44 and AIG44 in AsPC-1 cells, both with/without pre-incubation with C225 or NBMPR to demonstrate possible uptake mechanisms. Figure 2F shows the anti-proliferative effect of ACG44, AIG44 and CG44 on AsPC-1 cells determined through ^3^H-thymidine incorporation.

Studies were performed to ascertain the targeting efficacy of C225 and the functional activity of gemcitabine in the nanoconjugated form against various pancreatic cancer cell lines *in vitro*. The targeting experiments were performed in two ways to address the role of serum components on targeting efficacy; (i) the nanoconjugates were preincubated with cell growth media for 15 minutes before adding to the cells in tissue culture dishes; (ii) as synthesized nanoconjugates were directly added to the cells in tissue culture dishes. It is evident from the Figure 2A and Figure S2 that the addition of C225/IgG increases the absorbance of the GNPs followed by a red shift in the λ_max_. Subsequent addition of gemcitabine further shifts the λ_max_ while simultaneously increasing the absorbance, suggesting binding of both the components to the GNPs. Interestingly, preincubation with cell growth media decreases the absorbance of all the nanoconjugates probably due to the formation of a protein corona around the GNPs [46]. Additionally, TEM analysis showed efficient intracellular uptake of the ACG44 nanoconjugates in AsPC-1 cells (Figure 2B) and no significant aggregation of the particles in the presence of serum was observed (Figure S3) further supporting the formation of protein-corona after pre-incubation with the serum.

The effect of preincubation on the intracellular uptake of different nanoconjugates (AC4, ACG44, AI4 and AIG44) with AsPC-1 cells *in vitro* was determined. Uptake efficiency was determined by measuring the gold content in the cell pellet through INAA analysis (Figure 2C). It is evident from the Figure that AC4 and ACG44 are far more effective in targeting AsPC-1 cells than AI4 and AIG44. It is also interesting to note that cellular uptake of ACG44 is higher than AC4. This enhanced uptake of ACG44 could be due to the presence of gemcitabine, mediating uptake through nucleoside transporters (NTs), or due to an alternative path because of the available reactive surface area on gold nanoparticles. The non-specific uptake appears to be minimized after preincubation, which could be due to the coating of serum components on the available gold surface. Non-specific uptake of AI4 and AIG44 was completely inhibited by preincubation with the cell growth media. These data clearly suggest that C225 retains its specific targeting ability to EGFR-expressing cancer cells *in vitro*. The ability of C225 in the nanoconjugated form to effectively target EGFR over-expressing AsPC-1 cells was further confirmed by preincubation with C225. Preincubating AsPC-1 cells with C225 greatly diminished the uptake of ACG44, whereas there was no intracellular uptake of AIG44. These results further confirm that the endocytosis of ACG44 is via the EGFR pathway. Similarly, no difference in intracellular uptake was observed when cells were pre-treated with a nucleoside transporter blocker (NBMPR) [47]–[49], suggesting the absence of nucleoside transporter mediated uptake of the nanoconjugates (Figure 2E).

### Testing Functional Activity of Gemcitabine in the Nanoconjugated Form with Various Pancreatic Cancer Cell Lines in vitro

We utilized three different pancreatic cancer cell lines, AsPC-1, PANC-1 and MiaPaca-2, all having variable EGFR expression, to test whether the activity of gemcitabine had been retained in the nanoconjugated form. It is evident from the Figures that ACG44, AIG44 and CG44 (a no gold control containing gemcitabine and C225) substantially inhibited proliferation of pancreatic cancer cells in a concentration dependent manner as determined by ^3^H-thymidine assays. It is also apparent from the Figure that maximum inhibition of AsPC-1 cells was observed at the highest dose of both ACG44 and AIG44, which is comparable to the same dose of C225 and gemcitabine in the no gold control, CG44. A similar trend was observed with PANC-1 and MiaPaca-2 cells (Figure 2F and Figure S4). These results clearly demonstrate that gemcitabine retains its functional activity in the nanoconjugated form.

### Stability of the Nanoconjugates in Biological Fluids

It is important to test the stability of the nanoconjugates in biological fluids before *in vivo* applications. The stability of the nanoconjugates in terms of gemcitabine release was performed in phosphate buffered saline (PBS) and in mouse plasma. It is evident from the Figure 1F that the ACG44 nanoconjugates are very stable both in PBS (only 5% of bound gemcitabine is released over a period of 24 hrs) as well as in mouse plasma (∼12% of gemcitabine released over a period of 24 hrs). Furthermore, for future clinical application it is important to determine the stability of the nanoconjugates after long-term storage. To address this concern, we flash froze the nanoconjugates in liquid nitrogen and lyophilized them to a powder form. The powder was easily reconstituted in water, with no noticeable aggregation observed upon visual inspection. The solution was then tested for functional efficacy to inhibit the proliferation of AsPC-1 cells. Similar inhibition in proliferation of pancreatic cancer cells was observed by the reconstituted nanoconjugates, confirming the stability of the nanoconjugates under long-term storage conditions (Figure S5).

### Therapeutic Efficacy of the Nanoconjugates to Inhibit Tumor Growth in an Orthotopic Model of Pancreatic Cancer

**Figure 3:**
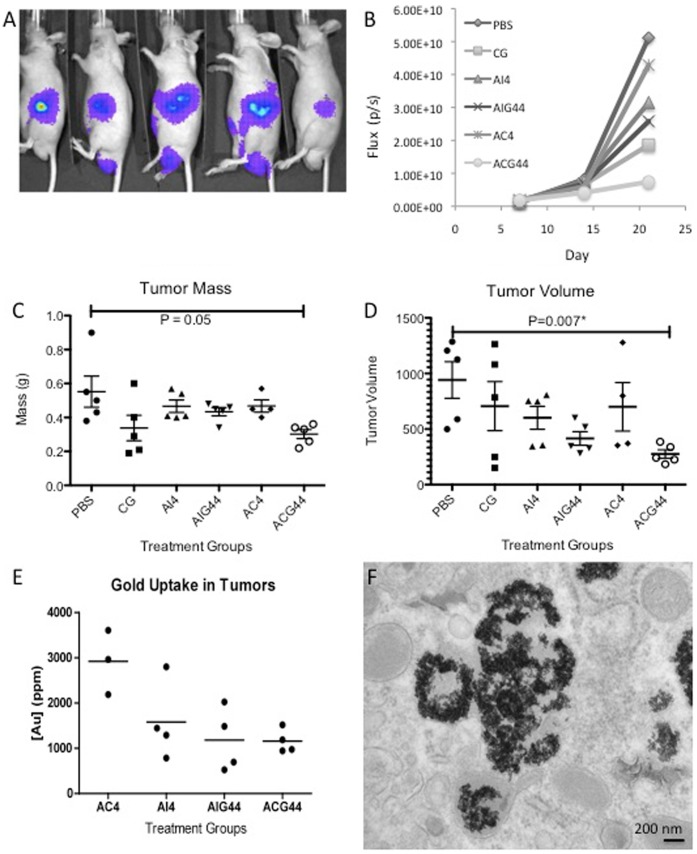
*In vivo* effects of gold nanoconjugates in an orthotopic model of pancreatic cancer. Figure 3A shows a representative bioluminescence image of 5 mice, 7 days after orthotopic implantation of AsPC-1 cells into the pancreas. Figure 3B is flux quantification from the bioluminescence imaging taken every 7 days. Figure 3C, 3D and 3E all show scatter plots of tumor analysis of each animal per group, post study termination. Figure 3C shows tumor mass, 3D shows tumor volume determined through caliper measurements and 3E shows total gold uptake in each tumor determined by INAA. Figure 3F is a TEM micrograph showing ACG44 conjugates in a cross section of tumor tissue.

**Figure 4:**
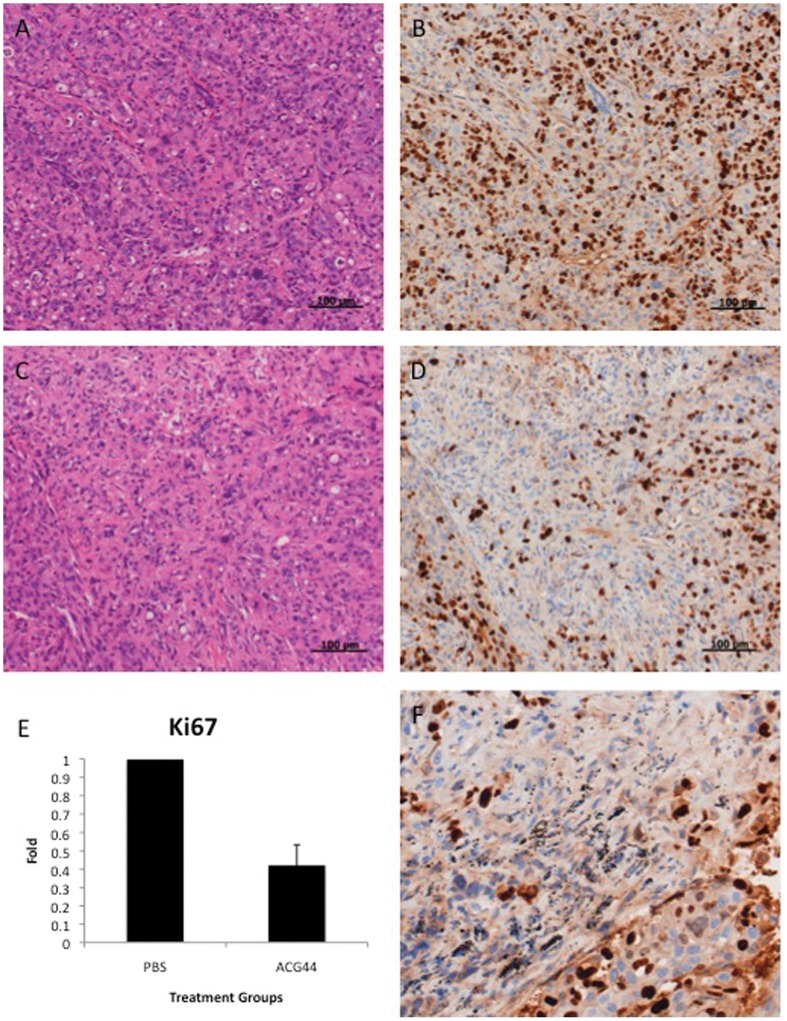
Immunohistochemistry Analysis of Tumors from the PBS and ACG44 groups. Figure 4A and 4B show representative images of H&E and Ki-67 stained tumor tissues, respectively, from the PBS treated group whereas Figure 4C and 4D show images of H&E and Ki-67 staining of tumor tissue from the ACG44 treated group. All images were taken with 20× magnification. Figure 4E is quantification of the Ki-67 positive proliferative nuclei shown in Figures 4B and 4D. Figure 4F is a tumor image of Ki-67 staining from the ACG44 treated group, taken at 100 X to show gold accumulation (black spots) at a high magnification.

To test the therapeutic efficacy of the nanoconjugates to inhibit tumor growth *in vivo*, we generated an orthotopic model of pancreatic cancer by implanting 2×10^6^ AsPC-1 cells directly into the pancreas of 4–6 week old nude male mice. To simulate an advanced stage of the disease, tumors were allowed to grow for 7 days before initiation of the treatment (Figure 3A). One week after the tumor cell implantation, the mice were imaged non-invasively through bioluminescence for tumor growth and randomized into 6 groups (n  = 5) before initiation of the treatment. The animals were injected in the intraperitoneal (i.p.) cavity thrice a week for the first week and twice for the next two weeks with 450 µg of gold nanoconjugates, 1.8 mg/kg of gemcitabine. The tumor progression was monitored non-invasively once a week with bioluminescence imaging as discussed above (Figure 3B). Bioluminescence measurements clearly demonstrate a significant reduction of tumor growth in ACG44 group as compared to the other groups. As expected, AC4 and AI4 had the least effect on decreasing the tumor growth while the AIG44 and CG44 groups had a moderate response due to the presence of gemcitabine. These observations were further confirmed by directly measuring tumor weight and volume after sacrificing the mice at the end of the experiment (Figure 3C and D). The ACG44 group showed the tightest cluster of data points as well as the highest therapeutic effect of all groups with an average tumor mass of 0.3 grams and tumor volume of 275 cm^3^ (0.55 g and 940 cm^3^ for the PBS group). Likewise, this therapeutic effect was also shown in the tumor gold content for the ACG44 group (Figure 3E). Further TEM analysis confirmed the uptake of ACG44 into the tumor tissue (Figure 3F), also show AIG44 internalization in Figure S7. Additionally, the biodistribution of gold in the liver, lungs, kidneys and spleen was also analyzed with INAA (Figure S6). As expected, the organs with the greatest uptake of gold were the spleen and the liver in all of the treatment groups. These results were further confirmed at the molecular level by quantifying the number of proliferating cells in different treatment groups using hematoxilin-eosin (H&E) and Ki-67 staining (Figure 4). Figure 4 exhibits H&E and Ki-67 for tumor tissue from the PBS (4A and B) and ACG44 groups (Figure 4C and D), respectively. The number of cancer cells as demonstrated by H&E staining are far less in ACG44 group than in the control PBS group. Furthermore, the number of proliferating cell nuclei as demonstrated by Ki-67 staining, is significantly decreased (∼60%) in the ACG44 group as opposed to the control PBS group (Figure 4E). Interestingly, treatment with ACG44 also revealed the presence of gold nanoparticles in the form of black specs inside the tumor tissue, suggesting better tumor penetration with the ACG44 nanoconjugates compared to the other groups. Additionally, it is evident from Figure 4 that the number of Ki-67 positive cells is greatly diminished surrounding the nanoparticles, suggesting a better therapeutic effect correlating with proximity to the nanoparticles. Taken together, these data indicate that the targeted delivery of a low dose of gemcitabine via gold nanoparticles can significantly inhibit the tumor growth in an advanced model of orthotopic pancreatic cancer *in vivo*.

## Discussion

Pancreatic cancer is one of the deadliest among human malignancies with no effective therapies currently available [11], [15]. Pancreatic cancer is typically detected in the late stage of the disease and the overall survival from this late diagnosis is commonly just a few months [1]. Surgery is the only option when detected early and gemcitabine is the front line chemotherapy for the advanced stage of the disease. However, chemotherapy with gemcitabine has only a limited therapeutic effect due to severe dose limiting toxicity. Thus, the overall survival is only 5–6 months. For this reason, effective therapeutic strategies are urgently required to combat this deadly disease. Therefore, any approach that will reduce the systemic toxicity and increase the efficacy of gemcitabine will have a significant impact on the therapeutic management of pancreatic cancer.

It has been recently recognized that nanotechnology has great potential to improve the quality of lives of cancer patients [22], [50], [51]. Major areas where nanotechnology can impact significantly in cancer are; i) Detection/diagnosis; (ii) Imaging and (iii) Therapeutics [52], [53]. Specifically, targeted drug delivery could significantly impact the therapeutic management of cancer by increasing the efficacy and reducing systemic toxicity of many chemotherapeutics. Development of a nanoparticle formulation to effectively target tumors is an area of active investigation. Among the inorganic nanomaterials, gold nanoparticles have generated considerable interest in various biomedical applications including targeting [27]. Several advantages that GNPs have over other nanomaterials are; (i) ease of synthesis; (ii) ease of characterization due to the presence of the SPR band; (iii) ease in binding of biomolecules such as peptides, proteins and antibodies exploiting the gold-thiol, gold-amine interactions and most importantly, (iv) biocompatibility compared to other inorganic nanomaterials currently being investigated [30], [54]–[57]. Previously we demonstrated that the targeted delivery of a low dose of gemcitabine using cetuximab bound to a gold nanoparticle in a “2 in 1” fashion resulted in significant inhibition of tumor growth at an early stage of an orthotopic model of pancreatic cancer [25]. EGFR is overexpressed in a number of human malignancies. The FDA approved cetuximab, an anti-EGFR antibody, for the use alone or in combination therapies to treat various malignancies. Therefore, the successful development of a targeted drug delivery system containing C225, gemcitabine and gold nanoparticles could be widely applicable to a variety of malignancies including pancreatic cancer. Recently, we defined the design criteria to effectively target pancreatic cancer cells in an orthotopic model of pancreatic cancer *in vivo* [24]. Based on this prior work, we report here the design of an optimized targeted drug delivery system that inhibits pancreatic tumor growth in an advanced stage of the disease in an orthotopic model.

The binding of C225 and gemcitabine to the gold nanoconjugates was demonstrated by an increase in absorbance and a red shift of the absorption maxima in the SPR band of gold nanoparticles. These findings were further confirmed by stability testing in 150 mM NaCl. Uncovered nanoparticles undergo rapid aggregation in the presence of high salt concentration. However, such aggregation is prevented through surface coverage of the gold nanoparticles by a combination of cetuximab and gemcitabine.

Gemcitabine, being a purine nucleoside requires nucleoside transporters, such as human equilibrative nucleoside transporters (hENT) or concentrative nucleoside transporters (hCNT) for intracellular uptake [58]–[61]. Therefore, ACG44 may be taken up by the cells either via EGFR endocytosis through active targeting with C225 or via nucleoside transporters (NTs). In general, nanoconjugates having C225 exhibit much higher cellular uptake compared to nanoconjugates having the non-targeting antibody, IgG. Furthermore, preincubating pancreatic cancer cells that overexpress EGFR, such as AsPC-1, with C225 significantly reduces the uptake of ACG44 nanoconjugates, while the uptake of AIG44 remains unaffected. Additionally, treatment with a NT inhibitor, NBMPR, does not reduce the uptake of either of the nanoconjugates. Together, these results support the conclusion that ACG44 enters into the cells through EGFR mediated endocytosis and that NTs do not contribute significantly to the uptake of the nanoconjugates. These findings could prove beneficial in combating gemcitabine resistance in cancer cells with a low expression of NTs. It is also important to note that preincubating the nanoconjugates in serum, decreases the uptake of both AC4 and ACG44, suggesting the adsorption of serum proteins blocks the available reactive surface on the gold particle and thereby increases the specificity of targeting. Likewise, preincubation of AI4 and AIG44 in serum reduced the intracellular uptake, presumably by blocking the available surface area on the gold particle that is involved in non-specific uptake. The fact that AI4 and AIG44 uptake is reduced to a non-detectable level, further confirms the targeting specificity of AC4 and ACG44.

Stability of the nanoconjugates under the physiological salt concentrations (150 mM NaCl) and in biological fluids (PBS and mouse plasma) demonstrates the suitability of these nanoconjugates for *in vivo* use. Only 12% of gemcitabine was released over a 24 hr period in mouse plasma. Previously, we demonstrated a similar amount of C225 released under various physiological environments [24].

Finally, the efficacy of the nanoconjugates *in vivo* in an aggressive orthotopic model of pancreatic cancer was demonstrated. It is important to note here, that treatments in many studies are typically initiated in this model on day 3 or 4 after tumor cell implantation in the pancreas. However, to simulate an advanced stage of the disease, we allowed the tumor cells to grow in the pancreas for 7 days before initiating the treatment. Typically, all the animals would die in a model like this within 3–4 weeks of implantation. However, our *in vivo* data clearly demonstrate that a low dose of gemcitabine delivered in a targeted fashion significantly reduced the tumor growth in this advanced stage model. This study highlights the potential of a gold nanoparticle based targeted drug delivery system to inhibit tumor growth in a orthotopic model of pancreatic cancer in advanced stage of the disease.

### Conclusion

In conclusion, we demonstrated that a low dose of gemcitabine delivered in the form of a targeted drug delivery system inhibits tumor growth in an advanced stage of an orthotopic model of pancreatic cancer. Using different cellular uptake path inhibitors we demonstrated that the uptake of the nanoconjugates is specific to the EGFR pathway. We also demonstrated significant stability of the nanoconjugates under different biological environments, as well as long-term stability of the nanoconjugates after lyophilization and storage. The potential impact of this study to inhibit pancreatic tumor growth at the advanced stage is significant, as no therapy is currently available for this deadly disease. Future work will focus on further improving the therapeutic efficacy, understanding the pharmacokinetics and combining active targeting with passive targeting.

## Supporting Information

Figure S1 **Representative absorption spectrums of GNP, AC4, AI4 and the addition of various loadings of gemcitabine.** Figure S1A represents the absorption spectra of ACG4X nanoconjugates, after incubation of AC4 with gemcitabine (X = 1, 2, 4, 6 and 8 µg/ml) for 1 h. Figure S1B represents the absorption spectra of AIG4X conjugates.
(TIFF)

Figure S2 **Absorption spectrum showing the role of pre-incubation with serum of AIG44.** Figure S2 depicts the absorbance spectrums of GNP, AI4 and AIG44 before and after pre-incubation with serum either 15 minutes at room temperature or 2 hrs at 37°C.
(TIFF)

Figure S3 **Transmission electron microscopy images showing the effect of pre-incubating with serum on conjugate shape and size.** Figure S3A is AC4 pre-incubated with serum. Figure S3B is AI4 pre-incubated with serum. Figure S3C is ACG44 pre-incubated with serum and Figure S3D is AIG44 pre-incubated with serum.
(TIFF)

Figure S4 ***In Vitro*** **effect of nanoconjugates on pancreatic cancer cell lines.** Figure S4A and S4B shows the anti-proliferative effect as determined by ^3^H-thymidine incorporation assay, of ACG44, AIG44 and CG44 on MiaPaCa-2 and Panc-1 cells, respectively.
(TIFF)

Figure S5 **Effect of lyophilizing the nanoconjugates on** ***in vitro*** **proliferation with AsPc-1 cells as determined by ^3^H-thymidine incorporation assay.**
(TIFF)

Figure S6 **Tissue distribution of the nanoconjugates in vital organs.** Cumulative gold uptake concentrations in the lung, kidney, liver and spleen. Shown in ppm and measured by INAA.
(TIFF)

Figure S7 ***In vivo*** **internalization of AIG44 in tumor tissue depicted with a TEM image.**
(TIFF)

Table S1 **Dynamic Light Scattering and Zeta Potential Analysis of all Nanoconjugates.**
(TIFF)
