# Supplementary figures and images for: Inhibiting the Growth of Pancreatic Adenocarcinoma In Vitro and In Vivo through Targeted Treatment with Designer Gold Nanotherapeutics
Source: PLoS One. 2013 Mar 6;8(3):e57522. doi: 10.1371/journal.pone.0057522 (PMC3590245; doi:10.1371/journal.pone.0057522)

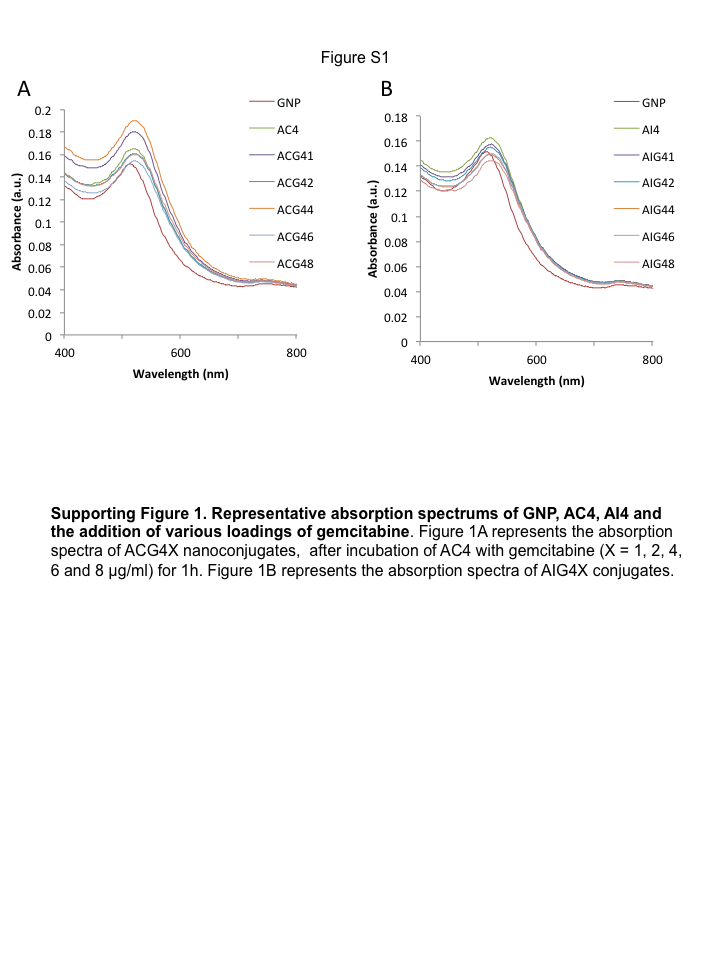

Supplement: Figure S1 — Representative absorption spectrums of GNP, AC4, AI4 and the addition of various loadings of gemcitabine. Figure S1A represents the absorption spectra of ACG4X nanoconjugates, after incubation of AC4 with gemcitabine (X = 1, 2, 4, 6 and 8 µg/ml) for 1 h. Figure S1B represents the absorption spectra of AIG4X conjugates. (TIFF) [file pone.0057522.s001.tiff]

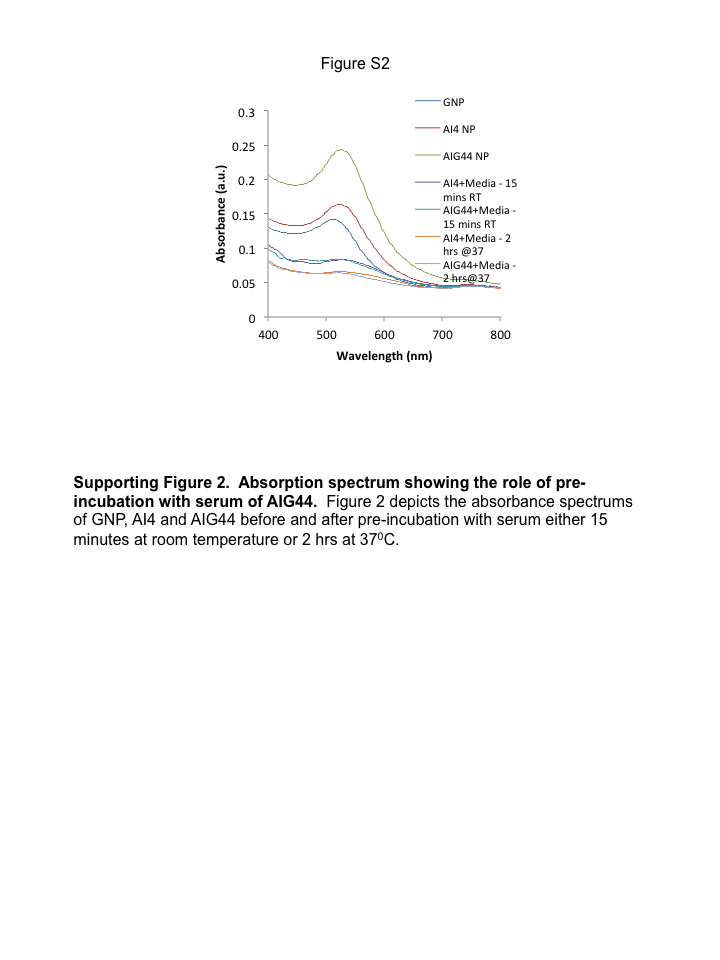

Supplement: Figure S2 — Absorption spectrum showing the role of pre-incubation with serum of AIG44. Figure S2 depicts the absorbance spectrums of GNP, AI4 and AIG44 before and after pre-incubation with serum either 15 minutes at room temperature or 2 hrs at 37°C. (TIFF) [file pone.0057522.s002.tiff]
